# Carbapenemase-Producing *Enterobacteriaceae* and Nonfermentative Bacteria, the Philippines, 2013–2016

**DOI:** 10.3201/eid2309.161237

**Published:** 2017-09

**Authors:** John Mark Velasco, Maria Theresa Valderama, Trent Peacock, Nirdnoy Warawadee, Kathyleen Nogrado, Fatima Claire Navarro, Domingo Chua, Srijan Apichai, Ruekit Sirigade, Louis R. Macareo, Brett Swierczewski

**Affiliations:** US Army Medical Directorate–Armed Forces Research Institute of Medical Sciences, Bangkok, Thailand (J.M. Velasco, M.T. Valderama, T. Peacock, N. Warawadee, K. Nogrado, S. Apichai, R. Sirigade, L.R. Macareo, B. Swierczewski);; V. Luna Medical Center, Quezon City, the Philippines (F.C. Navarro, D. Chua, Jr.)

**Keywords:** antimicrobial resistance, enteric infections, bacteria, multidrug resistance, New Delhi metallo-β-lactamase–producing, carbapenemase-producing, Enterobacteriaceae, nonfermentative bacteria, the Philippines, military treatment facility, carbapenem-resistant

## Abstract

During 2013–2016, we isolated *bla*_NDM_- and *bla*_VIM_-harboring *Enterobacteriaceae* and nonfermentative bacteria from patients in the Philippines. Of 130 carbapenem-resistant isolates tested, 45 were Carba NP–positive; 43 harbored *bla*_NDM_, and 2 harbored *bla*_VIM_. Multidrug-resistant microbial pathogen surveillance and antimicrobial drug stewardship are needed to prevent further spread of New Delhi metallo-β-lactamase variants.

Carbapenemase-producing *Enterobacteriaceae* can efficiently hydrolyze carbapenems and most β-lactam drugs. Since the identification of New Delhi metallo-β-lactamase-1 (NDM-1) in 2008 (*1*), there has been great concern regarding the spread of the Ambler class B metallo-β-lactamases (MBLs). Confirmed infections with MBL-positive bacteria are rarely identified in the Philippines, but *bla*_IMP_-harboring *Enterobacteriaceae* were reported in 2014 (*2*), an *Escherichia coli* (sequence type [ST] 131) isolate harboring *bla*_NDM-1_ was reported in 2014 (*3*), and 2 *Klebsiella pneumoniae* (ST626 and ST903) isolates harboring *bla*_NDM-1_ and *bla*_NDM-7_ genes were reported in 2016 (*4*).

We performed isolate identification and antimicrobial drug susceptibility testing by using the MicroScan WalkAway 40 plus System (Beckman Coulter, Brea, CA, USA) on 1,516 gram-positive and gram-negative isolates from patients admitted to various wards in the V. Luna Medical Center, a tertiary-care military hospital in Manila, the Philippines, during August 2013–April 2016. To better assess the distribution of carbapenem resistance and the underlying molecular mechanisms of resistance, we selected gram-negative isolates with imipenem or meropenem (or both) MICs of >8 μg/mL. We used microbroth dilution susceptibility testing (*5*) to select and verify 130 gram-negative nonrepeat isolates (i.e., each isolate was tested once) and then tested the isolates for carbapenemase production by using the Carba NP test as previously described (*6*). We tested all isolates with a Carba NP–positive result for *bla*_NDM_ and *bla*_KPC_ by using a multiplex real-time PCR assay as previously described (*7*,*8*); isolates with PCR-negative results were further tested, using the Xpert Carba-R PCR test with the GeneXpert IV System (both from Cepheid, Sunnyvale, CA, USA), for the presence of *bla*_NDM_, *bla*_KPC_, *bla*_VIM_, *bla*_IMP-1_, and *bla*_OXA-48_.

Of the 130 bacterial isolates tested, 45 (35%) had positive Carba NP test results and 43 (33%) harbored *bla*_NDM_; 25 (58%) of the *bla*_NDM_-carrying isolates were identified as *K. pneumoniae* (Technical Appendix Table). None of the isolates was positive for *bla*_KPC_. Two *Pseudomonas aeruginosa* isolates that had positive Carba NP test results were negative for *bla*_NDM_ and *bla*_KPC_ but positive for *bla*_VIM_. During the collection period, we also tested 8 environmental samples collected from the hospital’s neonatal intensive care unit and obstetrics and gynecology wards; 3 (38%) of the 8 isolates were positive for *bla*_NDM_ and identified as *K. pneumoniae* (Technical Appendix Table).

We report the identification of *bla*_NDM_-positive bacterial isolates in several genera of *Enterobacteriaceae* and nonfermentative bacteria in the Philippines. This finding is particularly significant because NDM-like enzymes have a broad range of activity against most β-lactam antimicrobial drugs and are often associated with serious clinical infections (*9*). A higher risk for plasmid-mediated transfer of NDM-1 exists through conjugation between different gram-negative bacterial strains (*10*), and NDM-1 can spread rapidly via nosocomial transmission or community-acquired infection. Furthermore, although limited in number, the environmental samples in this study were also positive for *bla*_NDM_, which suggests the possibility of nosocomial transmission and local circulation. 

We conducted multiplex real-time PCR testing only for *bla*_NDM_, *bla*_KPC_, *bla*_VIM_, *bla*_IMP-1_, and *bla*_OXA-48_ and did not investigate clonality; thus, further investigation into other carbapenemase genes should be conducted. In addition, further experiments should be performed to characterize the plasmids carrying the carbapenemase genes. Strengthening of multidrug-resistant microbial pathogen surveillance and antimicrobial drug stewardship is urgently needed to better characterize drug-resistance patterns and improve early detection and containment strategies in developing countries.

Technical Appendix Molecular resistance mechanisms of carbapenem-resistant clinical and environmental isolates from a tertiary-care military hospital, Manila, Philippines, August 2013–April 2016.
