# Supplementary material for: Carbapenemase-Producing Enterobacteriaceae and Nonfermentative Bacteria, the Philippines, 2013–2016
Source: Emerg Infect Dis. 2017 Sep;23(9):1597–8. doi: 10.3201/eid2309.161237 (PMC5572878; doi:10.3201/eid2309.161237)
Supplement: Technical Appendix — Molecular resistance mechanisms of carbapenem-resistant clinical and environmental isolates from a tertiary-care military hospital, Manila, Philippines, August 2013–April 2016. [file 16-1237-Techapp-s1.pdf]

Article DOI: https://doi.org/10.3201/eid2309.161237

Carbapenemase-Producing Enterobacteriaceae and Nonfermentative Bacteria, the Philippines, 2013–2016

Technical Appendix

Technical Appendix Table. Molecular resistance mechanisms of carbapenem-resistant clinical (n = 45) and environmental isolates (n = 3) from a tertiary-care military hospital in Manila, the Philippines, August 2013–April 2016*

Identification	Source	Organism	Carba NP	bla_{NDM}	bla_{KPC}	bla_{VIM}	Month	Year	Imipenem MIC (µg/mL)	Meropenem MIC (µg/mL)	Sex	Hospital ward
Patient isolates												
PH-0138–14	Blood	*K. pneumoniae*	Pos	Pos	Neg	ND	Nov	2013	>8	>8	M	Neonatal ICU
PH-0542–14	Soft tissue	*E. coli*	Pos	Pos	Neg	ND	Jun	2014	>8	>8	M	Pediatric
PH-0630–14	Wound	*K. pneumoniae*	Pos	Pos	Neg	ND	Jul	2014	>8	>8	F	Female Medical
PH-0631–14	Blood	*C. freundii*	Pos	Pos	Neg	ND	Jul	2014	>8	>8	M	Neurosurgery
PH-0756–14	Catheter	*K. pneumoniae*	Pos	Pos	Neg	ND	Aug	2014	>8	>8	F	Medical ICU
PH-0787–14	Endotracheal tip	*K. pneumoniae*	Pos	Pos	Neg	ND	Aug	2014	>8	>8	M	Medical ICU
PH-0837–14	Catheter	*K. pneumoniae*	Pos	Pos	Neg	ND	Sep	2014	>8	>8	F	Female Surgical
PH-0846–14	Catheter	*E. cloacae*	Pos	Pos	Neg	ND	Sep	2014	>8	>8	M	Male Medical Oncology
PH-0850–14	Urine	*K. pneumoniae*	Pos	Pos	Neg	ND	Sep	2014	>8	>8	F	Female Medical
PH-0873–14	Urine	*K. pneumoniae*	Pos	Pos	Neg	ND	Oct	2014	>8	>8	M	Surgical ICU
PH-0874–14	Wound	*Klebsiella sp.*	Pos	Pos	Neg	ND	Oct	2014	>8	>8	M	Female Medical
PH-0901–14	Wound	*C. freundii*	Pos	Pos	Neg	ND	Oct	2014	>8	>8	F	Female Surgical
PH-1037–14	Catheter	*K. pneumoniae*	Pos	Pos	Neg	ND	Dec	2014	>8	>8	F	Female Medical
PH-1076–14	Urine	*K. pneumoniae*	Pos	Pos	Neg	ND	Dec	2014	>8	>8	M	Medical ICU
PH-1078–14	Blood	*K. pneumoniae*	Pos	Pos	Neg	ND	Dec	2014	>8	>8	F	Female Medical
PH-1088–14	Endotracheal tip	*K. pneumoniae*	Pos	Pos	Neg	ND	Jan	2015	>8	>8	F	Medical ICU
PH-1093–14	Tracheal aspirate	*K. pneumoniae*	Pos	Pos	Neg	ND	Dec	2014	>8	>8	F	Female Medical
PH-1099–14	Endotracheal tip	*Acinetobacter sp.*	Pos	Pos	Neg	ND	Dec	2014	*	>8	M	Surgical ICU
PH-1115–14	Blood	*K. pneumoniae*	Pos	Pos	Neg	ND	Dec	2014	>8	>8	F	Female Medical
PH-1142–15	Blood	*K. pneumoniae*	Pos	Pos	Neg	ND	Jan	2015	>8	>8	F	Female Medical
PH-1143–15	Wound	*K. pneumoniae*	Pos	Pos	Neg	ND	Jan	2015	>8	>8	M	Neurology
PH-1150–15	Catheter	*E. cloacae*	Pos	Pos	Neg	ND	Jan	2015	>8	>8	M	Neurosurgery
PH-1159–15	Catheter	*K. pneumoniae*	Pos	Pos	Neg	ND	Jan	2015	>8	>8	M	Neurology
PH-1165–15	Urine	*K. pneumoniae*	Pos	Pos	Neg	ND	Jan	2015	>8	>8	M	Pulmonary Disease
PH-1166–15	Urine	*K. pneumoniae*	Pos	Pos	Neg	ND	Feb	2015	>8	>8	M	Surgical ICU
PH-1261–15	Blood	*C. freundii*	Pos	Pos	Neg	ND	Jul	2015	8	>8	M	Nephrology
PH-1263–15	Blood	*E. cloacae*	Pos	Pos	Neg	ND	Jul	2015	>8	>8	F	Neonatal ICU
PH-1265–15	Blood	*E. cloacae*	Pos	Pos	Neg	ND	Jul	2015	8	8	F	Neonatal ICU
PH-1266–15	Blood	*E. cloacae*	Pos	Pos	Neg	ND	Jul	2015	>8	>8	F	Neonatal ICU
PH-1270–15	Blood	*K. pneumoniae*	Pos	Pos	Neg	ND	Jan	2015	>8	>8	F	Female Medical

Identification	Source	Organism	Carba NP	bla_{NDM}	bla_{KPC}	bla_{VIM}	Month	Year	Imipenem MIC (μg/mL)	Meropenem MIC (μg/mL)	Sex	Hospital ward
PH-1279–15	Wound	*K. oxytoca*	Pos	Pos	Neg	ND	Jan	2015	8	>8	M	Neurology
PH-1280–15	Urine	*C. freundii*	Pos	Pos	Neg	ND	Jan	2015	>8	>8	M	Neurology
PH-1363–15	Wound	*P. aeruginosa*	Pos	Pos	Neg	ND	Sep	2015	>8	>8	M	Surgical ICU
PH-1379–15	Blood	*K. pneumoniae*	Pos	Pos	Neg	ND	Sep	2015	>8	>8	M	Medical ICU
PH-1384–15	Blood	*K. pneumoniae*	Pos	Pos	Neg	ND	Oct	2015	>8	>8	F	Medical ICU
PH-1394–15	Wound	*K. pneumoniae*	Pos	Pos	Neg	ND	Oct	2015	>8	>8	M	Surgical ICU
PH-1419–15	Urine	*K. pneumoniae*	Pos	Pos	Neg	ND	Oct	2015	>8	>8	F	Female Medical
PH-1477–15	Wound	*E. cloacae*	Pos	Pos	Neg	ND	Oct	2015	>8	>8	F	Medical ICU
PH-1478–15	Wound	*E. cloacae*	Pos	Pos	Neg	ND	Oct	2015	>8	>8	F	Female Medical
PH-1482–15	Wound	*K. pneumoniae*	Pos	Pos	Neg	ND	Oct	2015	>8	>8	F	Medical ICU
PH-1499–15	Wound	*E. cloacae*	Pos	Pos	Neg	ND	Oct	2015	>8	>8	F	Medical ICU
PH-1595–16	Blood	*E. cloacae*	Pos	Pos	Neg	ND	Jan	2016	>8	>8	F	Female Medical
PH-1641–16	Blood	*K. pneumoniae*	Pos	Pos	Neg	ND	Feb	2016	>8	>8	M	Neurology
PH-0745–14	Wound	*P. aeruginosa*	Pos	Neg	Neg	Pos	Aug	2014	8	>8	M	Male Surgical
PH-0905–14	Soft tissue	*P. aeruginosa*	Pos	Neg	Neg	Pos	Oct	2014	>8	>8	M	Post-anesthesia Care Unit
ATCC strains												
ATCC 2473		*K. pneumoniae*	Pos	Pos	Neg	ND	Apr	2016				
ATCC 1705		*K. pneumoniae*	Pos	Neg	Pos	ND	Apr	2016				
ATCC 1706		*K. pneumoniae*	Neg	Neg	Neg	ND	Apr	2016				
Environmental isolates bla_{NDM} positive (n = 3)												
E1	Laryngoscope	*K. pneumoniae*	Neg	Pos	Neg	ND	Nov	2014	>8	>8		Neonatal ICU
E3	Incubator	*K. pneumoniae*	Neg	Pos	Neg	ND	Nov	2014	>8	>8		Neonatal ICU
E7	Suction 1	*K. pneumoniae*	Neg	Pos	Neg	ND	Nov	2014	>8	>8		Neonatal ICU

*ICU, intensive care unit; ND, not done; Neg, negative; Pos, positive.
